# Screening of olfactory genes related to blood-feeding behaviors in *Culex pipiens quinquefasciatus* and *Culex pipiens molestus* by transcriptome analysis

**DOI:** 10.1371/journal.pntd.0010204

**Published:** 2022-02-07

**Authors:** Zhen-yu Gu, He-ting Gao, Qiao-jiang Yang, Meng Ni, Man-jin Li, Dan Xing, Tong-yan Zhao, Teng Zhao, Chun-xiao Li

**Affiliations:** 1 Anhui Medical University, Hefei, China; 2 State Key Laboratory of Pathogen and Biosecurity, Beijing Institute of Microbiology and Epidemiology, Beijing, China; 3 Beijing University of Chemical Technology, Beijing, China; Universita degli Studi di Pavia, ITALY

## Abstract

**Background:**

*Culex pipiens quinquefasciatus Say* (*Cx*. *quinquefasciatus*) and *Culex pipiens form molestus Forskal* (*Cx*. *molestus*) in the *Culex pipiens complex* group show considerable differences in host seeking, blood feeding, mating behavior and in vector competence. Blood-feeding mosquito behaviors are closely related to their olfactory gene expression and olfactory gene repertoire composition. Comparing olfactory genes between these two subspecies with significantly different blood-feeding behaviors can support further research on the molecular mechanism of the *Culex pipiens complex* olfactory sensory system, providing a new approach for determining candidate attractant or repellent compounds.

**Methods:**

Non-blood-feeding (NBF) and post-blood-feeding (PBF) olfactory system transcriptomes of the two subspecies were sequenced, and the biological functions of their differentially expressed genes were described by bioinformatics analysis. A quantitative polymerase chain reaction (qPCR) was applied to validate the RNA-seq data. The roles of particular olfactory receptors in *Cx*. *quinquefasciatus* blood-feeding behaviors were evaluated by RNAi.

**Results:**

Five, 7, 24, and 3 *Cx*. *quinquefasciatus*-specific OBPs, *Cx*. *molestus*-specific OBPs, *Cx*. *quinquefasciatus*-specific ORs and *Cx*. *molestus*-specific ORs were identified, respectively. The majority of selected ORs were consistent with the predicted transcriptome sequencing results after qRT-PCR validation. OR5 was expressed only in *Cx*. *quinquefasciatus*, and OR65 was the only gene upregulated after blood feeding in *Cx*. *molestus*. The blood-feeding rates of the OR5 and OR78 dsRNA groups were significantly lower (4.3%±3.1% and 13.3%±11.5%) than those of the enhanced green fluorescence protein (EGFP) group (64.5%±8.7%).

**Conclusion:**

Most OBPs and ORs were expressed in both subspecies but showed divergence in expression level. OR5 and OR65 might be species-specific expressed genes that regulate the olfactory behaviors of *Cx*. *quinquefasciatus* and *Cx*. *molestus*, respectively. The RNA interference of OR5 and OR78 could inhibit the blood-feeding behavior of *Cx*. *quinquefasciatus*, providing new targets for screening effective repellent compounds to control mosquito-borne diseases effectively and efficiently.

## Author summary

The transcriptomic gene expression of the olfactory tissues of *Cx*. *quinquefasciatus*the and *Cx*. *molestus* differ significantly. The majority of ORs and OBPs are expressed in both subspecies but are clearly differentiated in expression level. OR5 and OR65 may be species-specific olfactory genes expressed in *Cx*. *quinquefasciatus* and *Cx*. *molestus*, respectively. After the microinjection of OR5-dsRNA and OR78-dsRNA into female *Cx*. *quinquefasciatus* adults, the blood-feeding rate was significantly lower than that of the control group, suggesting that OR5 and OR78 are associated with the blood-feeding behavior of *Cx*. *quinquefasciatus*.

## 1. Introduction

*Culex pipiens quinquefasciatus Say (Cx*. *quinquefasciatus)*, which is widespread in tropical and subtropical regions, is an important pathogen vector that causes human diseases, such as West Nile Virus, St. Louis encephalitis, Sindbis fever, and equine encephalitis [1–3]. Infected mosquitoes transmit pathogens to other hosts, including humans, mammals, and birds [4], through blood feeding. Therefore, *Cx*. *quinquefasciatus* is an important species of mosquito vectors of public health concern.

Olfaction plays a vital role in the whole life cycle of mosquitoes, such as in their host-searching, mating, blood-feeding, and egg-laying processes [5–7]. The olfactory system of mosquitoes contains various olfactory proteins, such as odorant receptors (ORs), odorant-binding proteins (OBPs), and odorant-degrading enzymes (ODEs) [8–10]. There is a wide range of variation in the number of olfactory proteins corresponding to different mosquito species. Previous whole-genome analyses have annotated 79 ORs [11] and 69 OBPs [8] for *Anopheles gambiae*, 131 ORs and 111 OBPs [12] for *Aedes aegypti*, 82 ORs and 77 OBPs for *Aedes albopictus* [13], and 58 ORs and 58 OBPs for *Anopheles chinensis* [14]. Different olfactory proteins bind the same odor at different stages of mosquito development. For example, AaegOR10 and AaegOR9 are specifically expressed in adult and larval mosquitoes, respectively. Both of them are sensitive to skatole (3-methylindole). [15]. In fact, the mosquito OR gene repertoireare quite stable, which may be derived from a combination of functional divergence and the transcriptional modulation of orthologs. Even though the estimated gain/loss rates of ORs per million years are much lower than the overall level. [16],some OR subfamilies show evidence of positive selection (19 of 53 ORs in anopheline mosquito species) across the corresponding genus, suggesting potential functional divergence [17].

*Culex pipiens form molestus Forskal (Cx*. *molestus)* and *Cx*. *quinquefasciatus* belong to the same *Cx*. *pipiens complex* and can be distinguished morphologically through characteristics of the female wing, the abdominal terga, the male scutum and the phallosomes. [18,19] In physiological behaviors, *Cx*. *p*. *molestus* mates in confined spaces, feeds on mammals, and can lay eggs without a blood meal; additionally, females of this species cannot enter diapause in winter. *Cx*. *p*. *molestus* are known as underground mosquitoes because they invade basements, sewers, and pipes [20]. In contrast, *Cx*. *quinquefasciatus* mates in open spaces, feeds on birds and mammals, and must feed on blood to complete their reproductive cycle. Adult *Cx*. *quinquefasciatus* females diapause through the winter and swarm in open spaces. They breed on open waters, such as lakes and fetid ditches. Although important differences exist in blood-feeding habits and other physiological behaviors between *Cx*. *molestus* and *Cx*. *quinquefasciatus*, the molecular mechanisms behind these differences are still poorly understood [21].

We conducted this study to investigate the possible olfactory gene repertoires resulting in the blood-feeding behavior differences between the two species in the *Cx*. *pipiens complex* described above. First, we screened for differentially expressed OR and OBP genes in antennae between the two subspecies by transcriptome sequencing with *Cx*. *quinquefasciatus* whole genome as reference. Subsequently, the candidate genes were validated by a quantitative reverse-transcription polymerase chain reaction (RT-PCR) analysis. Finally, the dsRNA of the selected differentially expressed OR genes was injected into female adult mosquitoes to investigate whether they play a role in the blood-feeding behavior of *Cx*. *quinquefasciatus*. The screened olfactory genes involved in blood-feeding behavior would provide new targets for screening effective repellent compounds to control mosquito-borne diseases effectively and efficiently.

## 2. Materials and methods

### 2.1. Ethics statement

All procedures were conducted in accordance with the “Guiding Principles in the Care and Use of Animals” (GB/T 35892–2018) and were approved by the State Key Laboratory of Pathogen and Biosecurity Ethical Committee.

### 2.2. Mosquitoes and mice

Both *Cx*. *quinquefasciatus and Cx*. *molestus* are available from long-term reared laboratory strains. The breeding conditions were as follows: temperatures of 26±1°C, relative humidity of 70±5%, and a light:dark regime of 14 h:10 h. Adult mosquitoes were fed 8% sucrose water. Three to 5 days after eclosion, female *Cx*. *quinquefasciatus* specimens were fed mouse blood to lay eggs for breeding. The *Cx*. *molestus* specimens reproduced only autogenously. We selected female mosquitoes 3–5 days after eclosion for the experiments. Specific pathogen-free mice were used to test the host-locating ability of mosquitoes. There was no harm to the mice, as the number and durations of mosquito bites were strictly limited.

### 2.3. Transcriptome sequencing and bioinformatics analysis

Four groups were considered in this study: non-blood-feeding *Culex pipiens quinquefasciatus* (CqNBF), post-blood-feeding *Culex pipiens quinquefasciatus* (CqPBF), non-blood-feeding *Culex pipiens molestus* (CmNBF), and post-blood-feeding *Culex pipiens molestus* (CmPBF). Biological samples containing 50 antennae were set in triplicate for each group. Antennae RNA was extracted with TRIzol(Takara.9108), and libraries were constructed using the NEBNext Ultra RNA Library Prep Kit and finally sent to Beijing MacroMicro-test Biotechnology Co., Ltd. for transcriptome sequencing.

The raw data were filtered, and the clean reads were compared to the reference genome using HISAT2 software, reference file acquired from VectorBase (https://vectorbase.org/) [22]. Transcript assembly was performed using String Tie software [23] followed by annotation from databases such as P fam, SUPERFAMILY, Gene Ontology (GO), and the Kyoto Encyclopedia of Genes and Genomes (KEGG). Gene expression levels were quantitatively analyzed using the Counts tool in the subread software [24], and the corrected fragments per kilobase of transcript per million mapped reads (FPKM) values were combined [25,26]. A differential analysis was performed using DESeq2 software [27,28]. The screening criteria for differentially expressed genes (DEGs) in antennae were padj < 0.05 and |log2(fold change)| > 2. The screening criterion for different OBP and OR genes was padj < 0.05 due to their relatively low expression rates. Moreover, a chi-square test was used to confirm the DEseq2 results, comparing the gene expression difference between 2 paired groups like CqNBF and CqPBF. All RNA-Seq files are available from the Sequence Read Archive database (SRA accession number PRJNA757368, http://www.ncbi.nlm.nih.gov/bioproject/757368)

### 2.4. qPCR verification of identified olfactory genes

Genes fulfilled the following 2 criteria were selected, which should be detectable with relatively high expressions, and were statistically significantly different in at least two paired groups among CqNBF v.s.CmNBF,CqPBF v.s. CmPBF, CqPBF v.s.CqNBF and CmPBF v.s.CmNBF. Some genes that were eligible but had been validated in previous studies were excluded [29,30]. Finally we selected 14 olfactory genes and 2 housekeeping genes (RPL8 and 18S) to verify the accuracy of the results obtained herein. The primers were designed with Oligo Primer Analysis software version 4.0 (S1A Table). A real-time qPCR analysis was conducted in a 25-μL reaction system with a One Step SYBR PrimeScript RT-PCR Kit II (Cat# RR086A, Takara). The reaction conditions were set as follows: 94°C for 30 s; 94°C for 5 s, and 60°C for 30 s, repeated for 40 cycles. Three technical replicates were performed for each sample. The 2-ΔΔCT method was applied to calculate the relative gene expression [31,32].

### 2.5. RNAi of 3 selected OR genes and their effect on blood-feeding behavior

#### 2.5.1. Synthesis of dsRNA

Based on the transcriptome sequencing screening and PCR verification results, three OR genes that were specific/ highly expressed and significantly changed after blood-feeding in *Cx*. *quinquefasciatus*, were selected for follow-up RNAi analysis and tested for their effect on blood-feeding behaviors. These coding sequences (CDSs) were downloaded from the National Center for Biotechnology Information (NCBI) database, and the dsRNA primers were designed online from the E-RNAi website (http://e-rnai.dkfz.de). The OR5, OR78 and OR83 genes and EGFP dsRNA primers were synthesized (S1B Table). A MEGAscript RNAi Kit (Cat# AM1626, Thermo Fisher) was used to synthesize and purify the dsRNA. The dsDNA concentration was measured and then diluted to 600 ng/μl for the subsequent microinjections.

#### 2.5.2. Microinjection of adult mosquitoes

In the RNAi experiments, 5 groups were analyzed as follows: 3 treatment groups (injection of OR5/OR78/OR83-dsRNA), a negative control group (injection of EGFP-dsRNA), and a blank control group (no injection). Each group contained 30 mosquitoes, and all experiments were replicated 4 times. The sugar feeding of female *Cx*. *quinquefasciatus* specimens was halted for 10 hours before the experiments. The mosquitos were anesthetized with CO_2_ and then injected with 0.5 μL 600 ng/μl dsRNA in the side of their chest to reduce physical damage. The entire injection process was conducted at 4°C.

#### 2.5.3. Influence on blood-feeding behavior after RNAi

We proceeded with the blood-feeding behavior test 2 days postinjection. The supply of sugar water was halted for 8 hours before these tests. Specific pathogen-free (SPF) mice were fixed with clips and placed into cages for 30 minutes. The number of blood-feeding and nonblood-feeding female mosquitoes was counted, and then the blood-feeding rate of each group was calculated.

### 2.6. Statistical analysis

The 2-ΔΔCT method was used to calculate the relative olfactory gene expression rates [31,32]. DESeq2, which is suitable for small-scale samples and reduces the occurrence of false positives, was performed for the DEGs. A chi-square test was used to confirm the pairwise differences at the significance level of α = 0.05. All data were analyzed using SPSS software (version 21.0) and the R statistical language (version R 3.1.). The blood-feeding rate was calculated as the number of blood-feeding mosquitoes divided by the total number of tested mosquitoes multiplied by 100%.

## 3. Results

### 3.1. Different transcriptome expression of antennae between two subspecies

**Fig 1:**
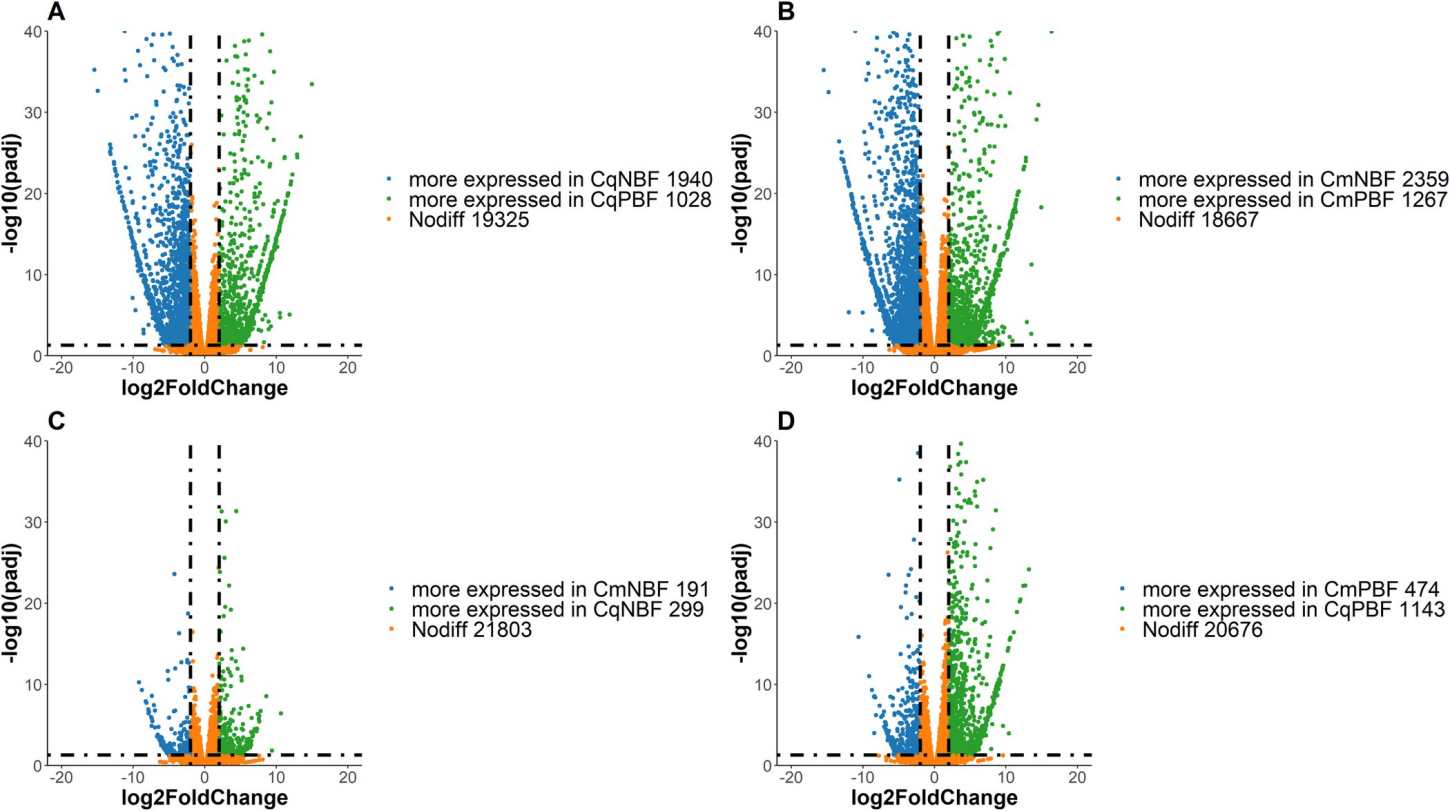
Volcano map of the differentially expressed antennal transcriptome genes (|log2FC|>2, padj<0.05). The green dots indicate significantly upregulated (more expressed in latter group) genes, the blue dots indicate significantly downregulated genes (more expressed in former group), and the orange dots represent nonsignificantly different genes. Fig 1A: Genes of *Cx*. *pipiens quinquefasciatus* before and after blood feeding; Fig 1B: genes of *Cx*. *pipiens molestus* before and after blood feeding; Fig 1C: genes of *Cx*. *pipiens quinquefasciatus* and *Cx*. *pipiens molestus* before blood feeding; and Fig 1D: genes of *Cx*. *pipiens quinquefasciatus* and *Cx*. *pipiens molestus* after blood feeding.

The principal component analysis results showed that 4 groups (two subspecies before and after blood-feeding) were obviously distanced, while replicates in the same group were quite stable in terms of environmental noise, suggesting significant differences between groups (S1 Fig). A total of 22,293 transcripts were obtained from the RNA-Seq results. We defined the transcripts with p<0.05 in groups comparison as significantly up/ down regulated and those with p<0.05 and log_2_FC > ±2.0 as DEGs. There were 20,037 transcripts identified from *Cx*. *quinquefasciatus*, in which 2,941 (14.68%) transcripts were significantly upregulated (more expressed in CqPBF) after blood feeding, including 1,028 transcripts with log_2_FC > 2.0; and 3,749 (18.71%) transcripts were significantly downregulated (more expressed in CqNBF), including 1,940 transcripts with log_2_FC < -2.0 (Fig 1A). Similarly, 20,018 transcripts were identified from *Cx*. *molestus*. There were 3,515 transcripts (17.56%, including 1,268 DEGs) that were significantly upregulated and 4,139 (20.68%, including 2360 DEGs) that were significantly downregulated after blood feeding (Fig 1B). There were 640 upregulated and 1,382 downregulated transcripts expressed in both *Cx*. *quinquefasciatus* and *Cx*. *molestus*.

Prior to blood feeding, the olfactory tissues of the two subspecies differed significantly in their expression of 1,923 transcripts, among which 299 and 192 DEGs differed between the CqNBF and CxNBF groups. (Fig 1C). After the mosquitoes fed on blood, 3,979 transcripts were significantly different between the two subspecies, including 1,143 transcripts with log_2_FC > 2.0 and 474 transcripts with log_2_FC < -2.0 as DEGs (Fig 1D). There were 123/51 transcripts with significantly higher/lower expression in *Cx*. *quinquefasciatus* than in *Cx*. *molestus*, no matter the mosquito fed on blood.

### 3.2. Different OBP transcripts between *Cx*. *quinquefasciatus* and *Cx*. *molestus*

Sixty-four and 66 OBP transcripts were sequenced from the *Cx*. *quinquefasciatus* and *Cx*. *molestus* transcriptomes, respectively. Most 59 OBPs were expressed in both subspecies. The number of *Cx*. *quinquefasciatus-*specific OBPs and *Cx*. *molestus*-specific OBPs were 5 and 7, respectively (S2 Fig). Alternative splicing generated different mRNA splice isoforms from one mRNA precursor by choosing different combinations of splice sites. And due to the existence of decommissioned base, different OBP genes transcripts such as CPIJ002105 and CPIJ002106 clustered together, which were both described as OBP58c. Based on the results of the DESeq2 analysis with a screening criterion of padj < 0.05, 22 OBP transcripts were filtered with significant differences in at least one paired comparison (Table 1).

**Table 1:** Significant differentially expressed OBP genes among the four compared groups.

Gene Name	Gene description	CqNBF v.s.CmNBF		CqPBF v.s.CmPBF		CqPBF v.s.CqNBF		CmPBF v.s.CmNBF	
		log2FC	Padj	log2FC	Padj	log2FC	Padj	log2FC	Padj
CPIJ001365	OBP2	0.195	0.680	0.062	0.863	**-1.246**	**<0.001**	**-1.077**	**<0.001**
CPIJ001730	OBP4	0.573	0.067	0.281	0.511	**-2.794**	**<0.001**	**-2.466**	**<0.001**
CPIJ002105	OBP58c	**0.888**	**<0.001**	**0.924**	**<0.001**	**-2.042**	**<0.001**	**-2.039**	**<0.001**
CPIJ002106	OBP58c	0.391	0.201	0.366	0.130	**-1.740**	**<0.001**	**-1.677**	**<0.001**
CPIJ002111	OBP50d	0.213	0.869	**-1.505**	**<0.001**	1.147	0.246	**2.906**	**<0.001**
CPIJ004634	OBPx	-0.209	0.908	0.920	0.108	**1.021**	**0.046**	-0.062	0.936
CPIJ007608	OBP5	-0.109	0.829	0.124	0.693	**-1.514**	**<0.001**	**-1.708**	**<0.001**
CPIJ007611	OBP56a	**0.739**	**0.030**	**0.884**	**<0.001**	**-1.438**	**<0.001**	**-1.546**	**<0.001**
CPIJ007617	OBP2	-0.008	0.990	0.247	0.438	**-1.836**	**<0.001**	**-2.050**	**<0.001**
CPIJ008793	OBP56a	**0.924**	**0.003**	0.223	0.364	**-2.381**	**<0.001**	**-1.642**	**<0.001**
CPIJ009568	OBP8	0.127	0.823	**1.945**	**<0.001**	**-0.897**	**<0.001**	**-2.674**	**<0.001**
CPIJ010789	OBP56a	**1.032**	**<0.001**	**6.870**	**<0.001**	**-1.082**	**<0.001**	**-6.885**	**<0.001**
CPIJ012717	OBP18	**-0.961**	**0.011**	**-3.542**	**<0.001**	**-1.717**	**<0.001**	**0.897**	**<0.001**
CPIJ012718	OBP56e	**0.641**	**0.005**	**1.831**	**<0.001**	**-2.243**	**<0.001**	**-3.401**	**<0.001**
CPIJ012719	OBP56d	0.163	0.635	**-0.710**	**<0.001**	**-2.183**	**<0.001**	**-1.272**	**<0.001**
CPIJ013976	OBP19a	**-2.431**	**<0.001**	**2.162**	**0.012**	**-2.236**	**<0.001**	**-6.809**	**<0.001**
CPIJ014525	OBP56a	-0.278	0.373	**-0.527**	**0.031**	**-0.811**	**<0.001**	**-0.522**	**0.017**
CPIJ016948	OBP56e	0.090	0.909	**2.786**	**<0.001**	-0.250	0.606	**-2.908**	**<0.001**
CPIJ016949	OBP12	**0.836**	**0.010**	0.300	0.281	**-2.396**	**<0.001**	**-1.822**	**<0.001**
CPIJ016951	OBP19a	**1.162**	**<0.001**	**3.929**	**<0.001**	**-1.807**	**<0.001**	**-4.536**	**<0.001**
CPIJ016965	OBP56e	-0.889	0.147	**4.231**	**<0.001**	**1.510**	**<0.001**	**-3.565**	**<0.001**
CPIJ016967	OBP56e	0.368	0.508	**4.565**	**<0.001**	0.484	0.185	**-3.674**	**<0.001**

The OBP genes are ordered by gene name. Padj refers to the adjusted P value, values of which under 0.05 are bolded. Log2Fc is short for log2 foldchange. Cq is short for *Culex pipiens quinquefasciatus*, and Cm is short for *Culex pipiens molestus*. NBF stands for non-blood-feeding, and PBF stands for post-blood-feeding. OBPx indicates an unnamed OBP.

**Fig 2:**
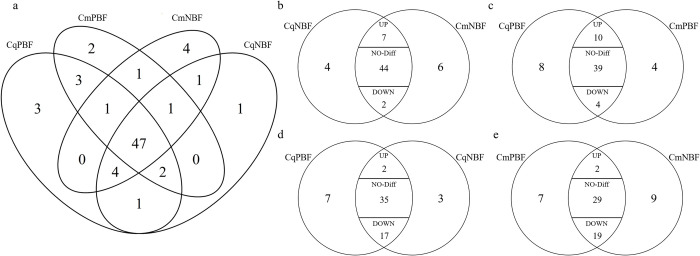
Venn diagrams of significantly differentially expressed OBP genes. **Fig 2A:** OBP genes intersections among 4 groups. Cq is short for *Culex quinquefasciatus*, and Cm is short for *Culex molestus*. NBF stands for non-blood-feeding, PBF stands for post-blood-feeding. **Fig 2B:** significantly differentially expressed OBP genes intersections between two subspecies before blood feeding. On the basis of the traditional venn diagram, we divided the overlapping Olive-shaped region into 3 sections “up, no-diff and down”. The X in “up” section means there were X genes significantly up-regulated when the two groups were compared. For example, there were 53(7+44+2) genes both expressed in CqNBF and CmNBF, in which 7 genes were significantly up-regulated, 2 genes down-regulated and 44 genes with no difference in expression level; **Fig 2C:** significantly differentially expressed OBP genes intersections between two subspecies after blood feeding; **Fig 2D:** significantly differentially expressed OBP genes intersections of *Cx*. *pipiens quinquefasciatus* before and after blood feeding; Fi**g 2E:** significantly differentially expressed OBP genes intersections of *Cx*. *pipiens molestus* before and after blood feeding.

The numbers and intersections of the OBP transcripts in the 4 groups are shown in Fig 2A There were 7 OR transcripts with significantly higher (up) and 2 with lower expression (down) in *Cx*. *quinquefasciatus* than in *Cx*. *molestus* before blood feeding. The CqNBF and CmNBF groups shared 44 OBPs with no difference (no-diff) in expression level (Fig 2B). Ten and 4 OBPs were significantly upregulated and downregulated in CqPBF compared with CmPBF, respectively (Fig 2C). Five transcripts of OBP58 (CPIJ002105), OBP56 (CPIJ007611/CPIJ010789/CPIJ012718) and OBP19 (CPIJ016951) were significantly upregulated in *Cx*. *quinquefasciatus* compared to in *Cx*. *molestus*, regardless of whether the mosquitoes fed on blood; OBP18 was expressed significantly less in *Cx*. *quinquefasciatus* than in *Cx*. *molestus* (Table 1).

Nineteen OBP transcripts were significantly differentially expressed after blood feeding in *Cx*. *quinquefasciatus* (Fig 2D). Twenty-one OBP transcripts were significantly differentially expressed after blood feeding in *Cx*. *molestus* (Fig 2E). Among them, 18 OBP transcripts were coregulated in both subspecies after blood feeding (Table 1).

### 3.3. Differential OR transcripts between *Cx*. *quinquefasciatus* and *Cx*. *molestus*

There were 124 and 103 OR transcripts sequenced from the *Cx*. *quinquefasciatus* and *Cx*. *molestus* transcriptomes in this study, respectively. Most 100 ORs were expressed in both subspecies. The number of *Cx*. *quinquefasciatus*-specific ORs and *Cx*. *molestus*-specific ORs were 24 and 3, respectively (S3 Fig). Several subgroups were formed such as CPIJ012950 and CPIJ012952, which were both described as OR94b. Based on the DESeq2 analysis results, 20 OR transcripts with significant differences in at least one paired group comparison were filtered with padj < 0.05 (Table 2).

**Table 2:** Significantly expressed ORs among the four compared groups.

Gene Name	Gene description	CqNBF v.s.CmNBF		CqPBF v.s.CmPBF		CqPBF v.s.CqNBF		CmPBF v.s.CmNBF	
		log2FC	Padj	log2FC	Padj	log2FC	Padj	log2FC	Padj
CPIJ000541	OR1	1.329	0.128	**6.037**	**0.002**	**-2.120**	**0.001**	**-6.794**	**<0.001**
CPIJ000545	OR5	1.799	0.175	4.155	0.225	**-2.451**	**0.025**	-4.777	0.056
CPIJ002698	OR83c	1.021	0.057	**2.224**	**<0.001**	**-0.983**	**0.006**	**-2.151**	**<0.001**
CPIJ002700	OR83c	0.904	0.176	**1.901**	**0.050**	-1.135	0.052	**-2.109**	**0.007**
CPIJ004152	OR13a	0.492	0.797	**6.439**	**<0.001**	0.394	0.751	**-5.525**	**0.007**
CPIJ004160	OR13a	**2.034**	**0.002**	**6.579**	**<0.001**	0.265	0.598	**-4.244**	**0.002**
CPIJ004161	OR13a	1.190	0.136	**2.927**	**0.001**	-0.624	0.329	**-2.327**	**0.019**
CPIJ004162	OR36	**1.549**	**<0.001**	**2.350**	**<0.001**	-0.291	0.477	**-1.055**	**0.016**
CPIJ004987	OR83c	0.663	0.392	1.550	0.152	**-1.412**	**0.005**	**-2.267**	**0.009**
CPIJ004988	OR83c	0.624	0.585	**3.970**	**0.002**	-0.938	0.184	**-4.251**	**0.001**
CPIJ005662	ORx	**1.338**	**0.013**	**2.950**	**<0.001**	**-1.095**	**0.045**	**-2.672**	**<0.001**
CPIJ005374	ORx	**2.130**	**<0.001**	**2.263**	**<0.001**	-0.324	0.378	-0.419	0.575
CPIJ005920	OR83c	0.756	0.224	1.980	0.114	**-2.110**	**<0.001**	**-3.302**	**<0.001**
CPIJ006217	OR65	-2.346	0.396	-1.297	0.334	3.224	0.073	**2.207**	**0.049**
CPIJ008779	OR83	**1.302**	**0.021**	1.450	0.122	**-2.192**	**<0.001**	**-2.292**	**<0.001**
CPIJ009573	OR7	0.072	0.924	-0.156	0.627	0.400	0.315	**0.665**	**0.001**
CPIJ012950	OR94b	**3.922**	**<0.001**	**6.867**	**<0.001**	**-1.333**	**0.021**	-4.234	0.156
CPIJ012952	OR94b	**2.384**	**0.010**	**6.720**	**<0.001**	-1.093	0.124	**-5.381**	**0.019**
CPIJ013644	OR110	-1.918	0.181	**-1.679**	**0.013**	**2.693**	**0.015**	**2.474**	**<0.001**
CPIJ020302	OR83c	-0.116	0.948	**5.385**	**0.004**	0.189	0.875	**-5.276**	**0.002**

The OR genes are ordered by gene name. Padj refers to the adjusted P value, values of which under 0.05 are bolded. Log2Fc is short for log2 foldchange. Cq is short for *Culex quinquefasciatus*, and Cm is short for *Culex molestus*. NBF stands for non-blood-feeding, and PBF stands for post-blood-feeding. ORx indicates an unnamed OR.

**Fig 3:**
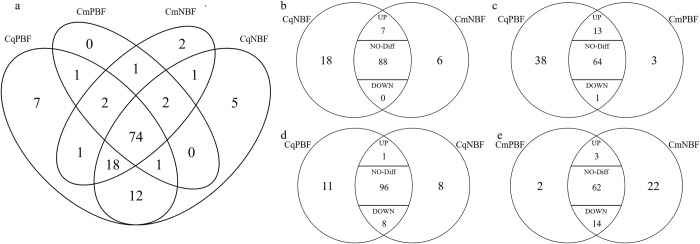
Venn diagrams of significantly differentially expressed OR genes. **Fig 3A: OR genes intersections among 4 groups.** Cq is short for *Culex quinquefasciatus*, and Cm is short for *Culex molestus*. NBF stands for non-blood-feeding, and PBF stands for post-blood-feeding. **Fig 3B:** significantly differentially expressed OR genes intersections between two subspecies before blood feeding: On the basis of the traditional venn diagram, we divided the overlapping Olive-shaped region into 3 sections “up, no-diff and down”. The X in “up” section means there were X genes significantly up-regulated when the two groups were compared. For example, there were 95(7+88+0) genes both expressed in CqNBF and CmNBF, in which 7 genes were significantly up-regulated, 0 genes down-regulated and 88 genes with no difference in expression level; **Fig 3C:** significantly differentially expressed OR genes intersections between two subspecies after blood feeding; **Fig 3D:** significantly differentially expressed OR genes intersections of *Cx*. *pipiens quinquefasciatus* before and after blood feeding; **Fig 3E:** significantly differentially expressed OR genes intersections of *Cx*. *pipiens molestus* before and after blood feeding.

The numbers and intersection of the OR transcripts in the 4 groups were shown in Fig 3A. There were 7 OR transcripts with significantly higher expression in *Cx*. *quinquefasciatus* than in *Cx*. *molestus* before blood feeding (Fig 3B). Thirteen and 1 ORs were significantly upregulated and downregulated in CqPBF compared with CmPBF, respectively (Fig 3C). Six OR transcripts were significantly higher in *Cx*. *quinquefasciatus* before and after blood feeding than in *Cx*. *molestus*, respectively (Table 2).

The numbers of OR transcripts in CqPBF that were significantly upregulated and downregulated compared with those in CqNBF were 1 and 8, respectively (Fig 3D). Three and 14 OR transcripts were significantly differentially expressed after blood feeding in *Cx*. *molestus*. (Fig 3E); among them, OR110 was an upregulated transcript. Six transcripts, including OR83 (CPIJ008779, Not the OR obligatory co-receptor OR83b CPIJ009573), were coregulated at lower levels in both subspecies after blood feeding (Table 2)

### 3.4. Quantitative PCR validation

**Fig 4:**
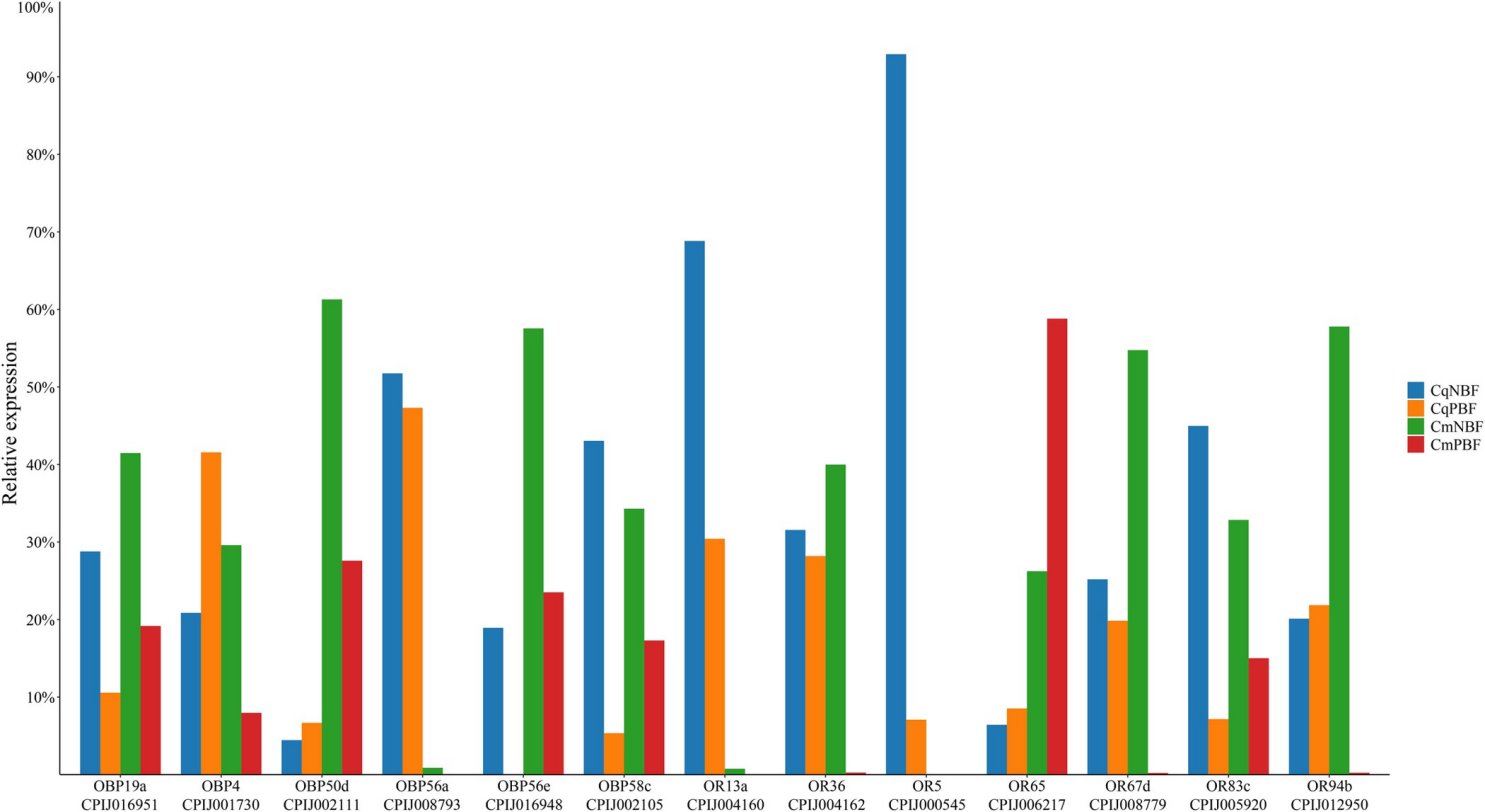
Quantitative PCR validation results for significantly different olfactory genes. The column represents the relative expression ratio; the total of the 4 columns for each individual gene should total 100%.

The results of the real-time fluorescence quantitative PCR validation of olfactory-related genes are shown in Fig 4. OR5 was expressed only in *Cx*. *quinquefasciatus*, and OR65 was the only gene upregulated after blood feeding in *Cx*. *molestus*. Among the selected olfactory transcripts, the majority were consistent with the predicted transcriptome sequencing results, except for OBP4, OBP50d, OR94b and OR13a. The up or down regulation trend between CqNBF and CqPBF was reversed in the former 3 genes. OR13a was not detected in CmPBF, which led to inconsistent results compared to those obtained from the transcriptome analysis.

### 3.5. Effect of three selected RNA-interfering ORs on blood-feeding behavior

**Fig 5:**
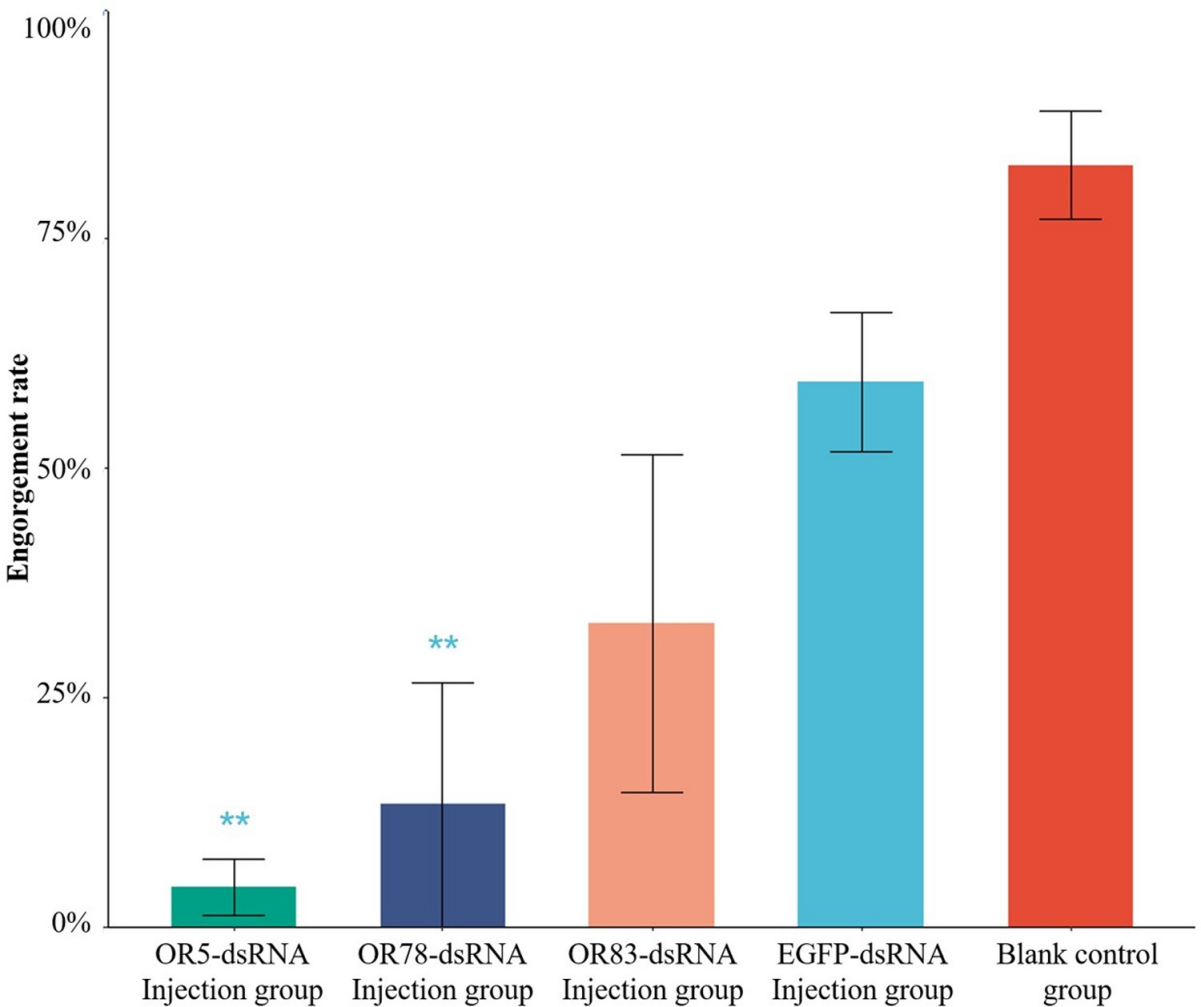
The blood-feeding rates of *Cx*. *pipiens quinquefasciatus* following the injections of different dsRNAs. The bar length indicates the standard deviation. chi-square test was used to compare the pairwise differences at the significance level of α = 0.05. Two asterisks (**) represent p < 0.01, and one asterisk (*) represents p < 0.05 compared to the EGFP control. No difference was found between the EGFP control and blank control.

The results of the blood feeding behaviors of female *Cx*. *quinquefasciatus* mosquitoes tested after the injection of different dsRNAs are shown in Fig 5; No significant difference was found between the two control groups (64.5% ± 8.7% and 82.4% ± 9.3%), indicating that the effect of the physical damage caused by the injections was limited. The blood-feeding rates of the mosquitoes that received OR5-dsRNA (4.3% ± 3.1%) and OR78-dsRNA (13.3%±11.5%) injections were significantly lower than those of the mosquitoes in the EGFP-dsRNA control groups (P<0.05), while no significant difference was found for OR83-dsRNA (33.1%±26.0%), suggesting that OR5 and OR78 may affect *Cx*. *quinquefasciatus* blood-feeding behaviors.

## 4. Discussion

Compared to the 180 ORs [33] and 109 OBPs [34,35] annotated in the *Cx*. *quinquefasciatus* genome, only two-thirds of these OR and OBP transcripts were sequenced in our study. The explanations for these results are presumed to be various. Some of these olfactory genes were either not expressed in antennae tissue or were not expressed during the experimental stage. Methodological issues like sequencing detectable threshold may lead to the low expression rates of undetected genes. Relatively limited 3 biological replicates could affect the power to detect differentially expressed genes.

The majority of the OR and OBP transcripts were expressed in both subspecies, since they are in the same mosquito complex; however, divergence was still observed in the olfactory gene repertoire number and expression level. In summary, we focused on olfactory genes that were both differentially expressed between subspecies and highly homologous to known functional genes in other species. For example, OBP18 was expressed at significantly lower levels in *Cx*. *quinquefasciatus* compared to in *Cx*. *molestus*. A previous study showed that *Culex pipiens pallens*, another subspecies in the *Culex pipiens complex*, which needed a blood meal before egg-laying like *Cx*. *quinquefasciatus*, also had significantly lower OBP18 expression than *Cx*. *molestus* in olfactory tissues [30], suggesting that OBP18 may be associated with ecological and biological differences that exist within the *Culex pipiens complex*. Whether this differentiation affects the autogenous or host preference behaviors needs to be explored further.

A phylogenetic tree showing the olfactory repertoire interspecific relations was shown in S4 Fig. Several genes were considered in additional species of the Anophelinae and Culicinae lineages because of their identified function on blood-feeding behaviors. Highly homologous olfactory proteins in different mosquito species may respond to the same host odor and mosquito oviposition pheromone [36]. For example, OBP1 showed a strong binding ability to methylindole both in *Culex quinquefasciatus* [37] and in *Anopheles gambiae* [38], and OR2 can specifically bind to indoles in *Culex quinquefasciatus* and *Aedes aegypti* [33]. In our study, the OBP2 transcripts (CPIJ001365 and CPIJ007617) were jointly downregulated after blood feeding in both subspecies, showing homology with a 48.4% amino acid identity with AgamOBP2 in *Anopheles gambiae*. A previous study found that AgamOBP2 is associated with the feeding and reproductive behaviors of female mosquitoes [39]. It has been speculated that OBP2 may correspond to similar feeding and reproductive functions in *Cx*. *quinquefasciatus* and *Cx*. *molestus*. In addition, OR1 was also downregulated in both subspecies after blood feeding and shared 36.9% of its amino acid identity with AgamOR1 of *Anopheles gambiae*, which is sensitive to human sweat [9,40]. It has been hypothesized that the CquiOR1 transcript of *Cx*. *quinquefasciatus* and the CmolOR1 transcript of *Cx*. *molestus* may also be related to host locating.

Genes with distinct expression differences may facilitate breakthroughs in distinguishing the blood-feeding behavior of different mosquito species. The OR65 expression levels were diverse in the *Culex pipiens complex*. OR65 was significantly high after blood feeding in *Cx*. *molestus* mosquitoes, while it was extremely low in *Cx*. *quinquefasciatus* and *Culex pipiens pallens* [30]. As the AgOR65 transcript of *Anopheles gambiae*, which was clustered with CmolOR65 in the phylogenetic tree, responded strongly to 2-ethylphenol volatilized from animal urine [40], it was hypothesized that *Cx*. *molestus* might show different blood-feeding behavior compared to other subspecies by sensing particular odor chemicals with CmolOR65; this hypothesis requires further study.

Mosquitoes reduced olfactory activity after blood-feeding and tuned down the olfactory response to control energy consumption for midgut blood digestion and ovary development. ORs delay reactivation after the end of blood digestion to search for egg-laying sites [41]. The RNAi experiments conducted herein confirmed that OR5 and OR78 were associated with blood-feeding behaviors. Although we were not able to completely silence OR5 and OR78, probably because of the high level of transcription, the partial knockdown clearly affected blood-feeding behaviors. The level of OR83 transcript reduction achieved by our RNAi treatments may not be high enough to affect blood-feeding rate with remarkable sensitivity. OR5 was detected only in *Cx*. *quinquefasciatus* but not in *Cx*. *molestus*. A previous study found that OR5 in *Cx*. *quinquefasciatus* (CquiOR5) is one of the receptors that binds mosquito-repellent substances, such as linalool and p-menthane-3,8-diol (p-menthane-3,8-diol PMD) [42]. The OR5 transcript of *Anopheles gambiae* (AgOR5) reacts strongly to the chemical substance 2,3-butanedione derived from the human skin microbiome [40]. Highly homologous olfactory protein could bind different chemicals and lead to different effects, which might be due to synergistic or antagonistic effects between multiple proteins, or to external reasons such as concentration. One particular odorant could elicit strong responses to several receptors. Reciprocally, one receptor could respond to several odorants and lead to diverse olfactory-guided behaviors. No other studies have mentioned the relevance of OR78 to certain mosquito behaviors. In our study, significant changes in the blood feeding rate observed when OR78 was interrupted indicated that CquiOR78 may regulate blood-feeding behaviors in *Cx*. *quinquefasciatus*. To explain the relationships between certain odorants and receptors in guiding blood-feeding behaviors, further research should focus on the functional characterization of entire receptor repertoires [43].

This study of the diverse olfactory genes of *Cx*. *pipiens molestus* and *Cx*. *pipiens quinquefasciatus* lays the foundation for further elucidating the olfactory sensory system molecular mechanism behind the differences of blood-feeding habits and other physiological behaviors. Through specific and targeted binding capacity assay, the screened olfactory genes would provide new targets for attractant and repellent candidate compounds to control mosquito-borne diseases effectively and efficiently.

## Supporting information

S1 Fig Principal component analysis of four groups of samples. Cq is short for *Culex quinquefasciatus*, and Cm is short for *Culex molestus*. NBF stands for non-blood-feeding, and PBF stands for post-blood-feeding.
(TIF)

S2 Fig Phylogenetic analysis of the OBP gene of *Cx*. *quinquefasciatus* and *Cx*. *molestus*. Phylogenetic tree was constructed by the maximum likelihood (ML) method using Molecular Evolutionary Genetics Analysis (MEGA) 7.0 software, with 1,000 bootstrap replicates.
(TIF)

S3 Fig Phylogenetic analysis of the OR gene of *Cx*. *quinquefasciatus* and *Cx*. *molestus*. Phylogenetic tree was constructed by the maximum likelihood (ML) method using Molecular Evolutionary Genetics Analysis (MEGA) 7.0 software, with 1,000 bootstrap replicates.
(TIF)

S4 Fig Phylogenetic analysis of highly homologous olfactory genes in different mosquito species. Aga stands for *Anopheles gambiae*, *Cqui* stands for *Cx*. *quinquefasciatus*, *Aag* stands for *Aedes aegypti*. Phylogenetic tree was constructed by the maximum likelihood (ML) method using Molecular Evolutionary Genetics Analysis (MEGA) 7.0 software, with 1,000 bootstrap replicates.
(TIF)

S1 Data OR and OBP with all the accession numbers (gene name) and gene description. (CSV)

S1 Table Primers designed and used in this study. **Table A:** The qPCR primers designed to verify olfactory genes; **Table B**: The primers used to synthesize dsRNA.
(DOCX)
