# Supplementary figures and images for: Screening of olfactory genes related to blood-feeding behaviors in Culex pipiens quinquefasciatus and Culex pipiens molestus by transcriptome analysis
Source: PLoS Negl Trop Dis. 2022 Feb 7;16(2):e0010204. doi: 10.1371/journal.pntd.0010204 (PMC8853563; doi:10.1371/journal.pntd.0010204)

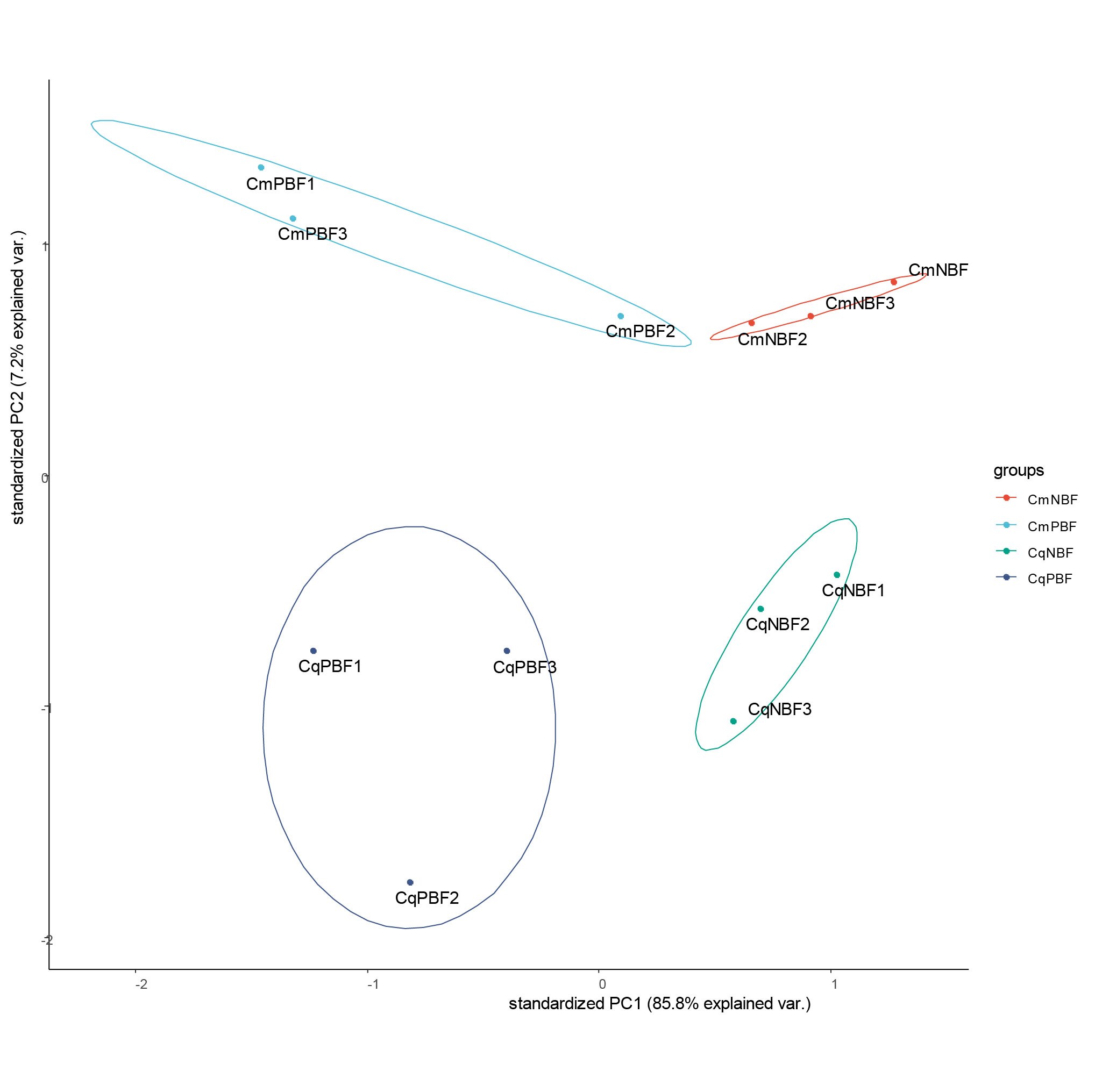

Supplement: S1 Fig — Cq is short for Culex quinquefasciatus, and Cm is short for Culex molestus. NBF stands for non-blood-feeding, and PBF stands for post-blood-feeding. (TIF) [file pntd.0010204.s001.tif]

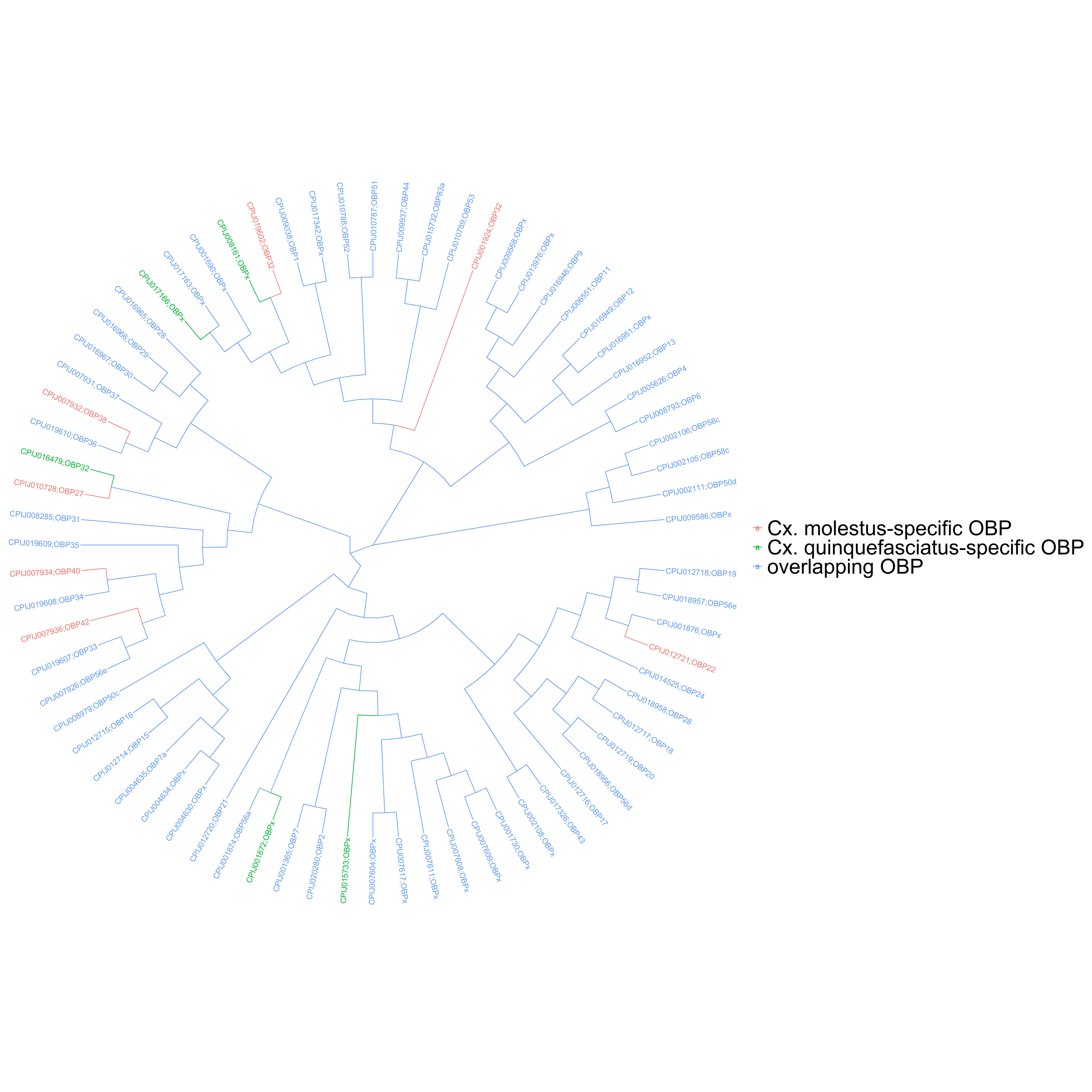

Supplement: S2 Fig — Phylogenetic tree was constructed by the maximum likelihood (ML) method using Molecular Evolutionary Genetics Analysis (MEGA) 7.0 software, with 1,000 bootstrap replicates. (TIF) [file pntd.0010204.s002.tif]

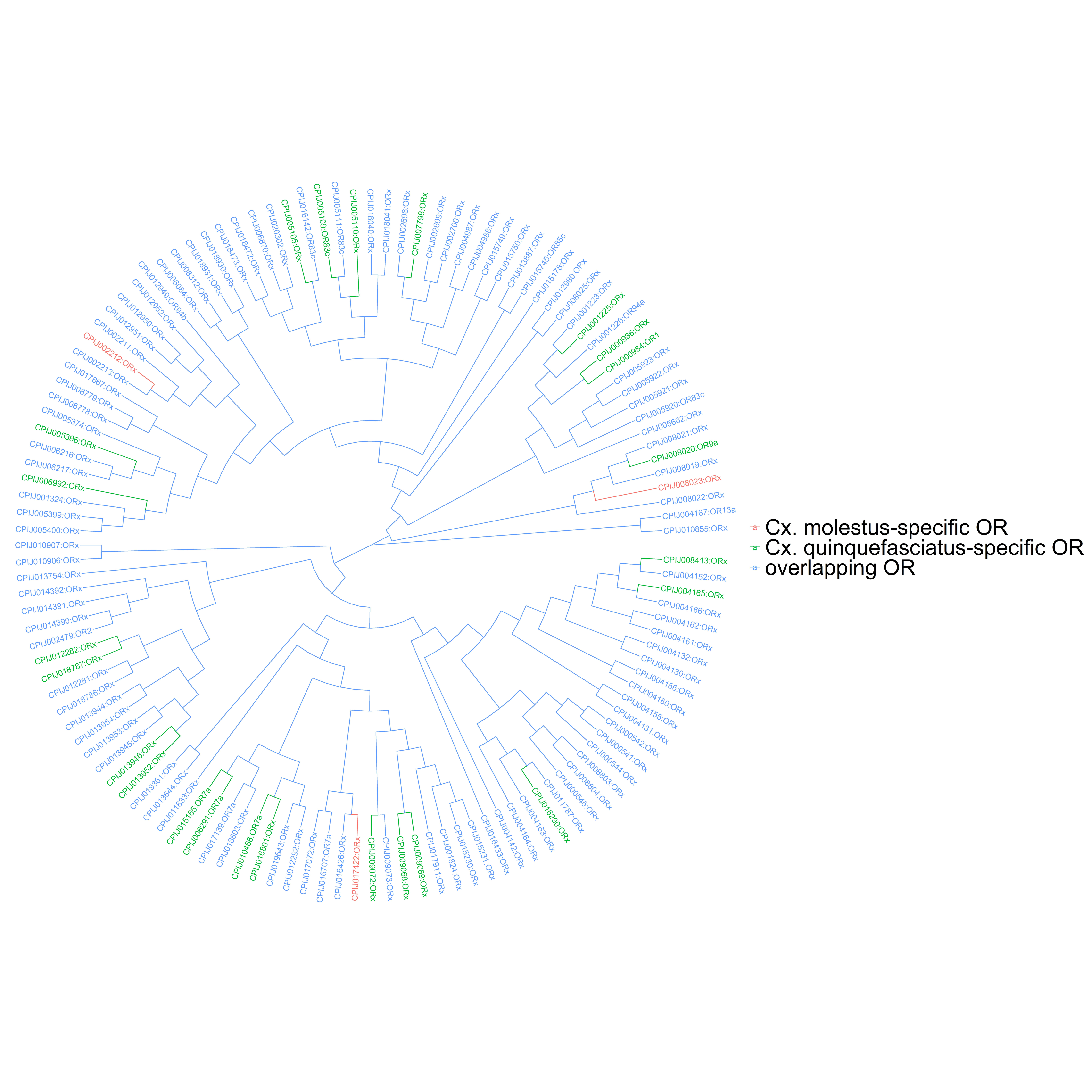

Supplement: S3 Fig — Phylogenetic tree was constructed by the maximum likelihood (ML) method using Molecular Evolutionary Genetics Analysis (MEGA) 7.0 software, with 1,000 bootstrap replicates. (TIF) [file pntd.0010204.s003.tif]

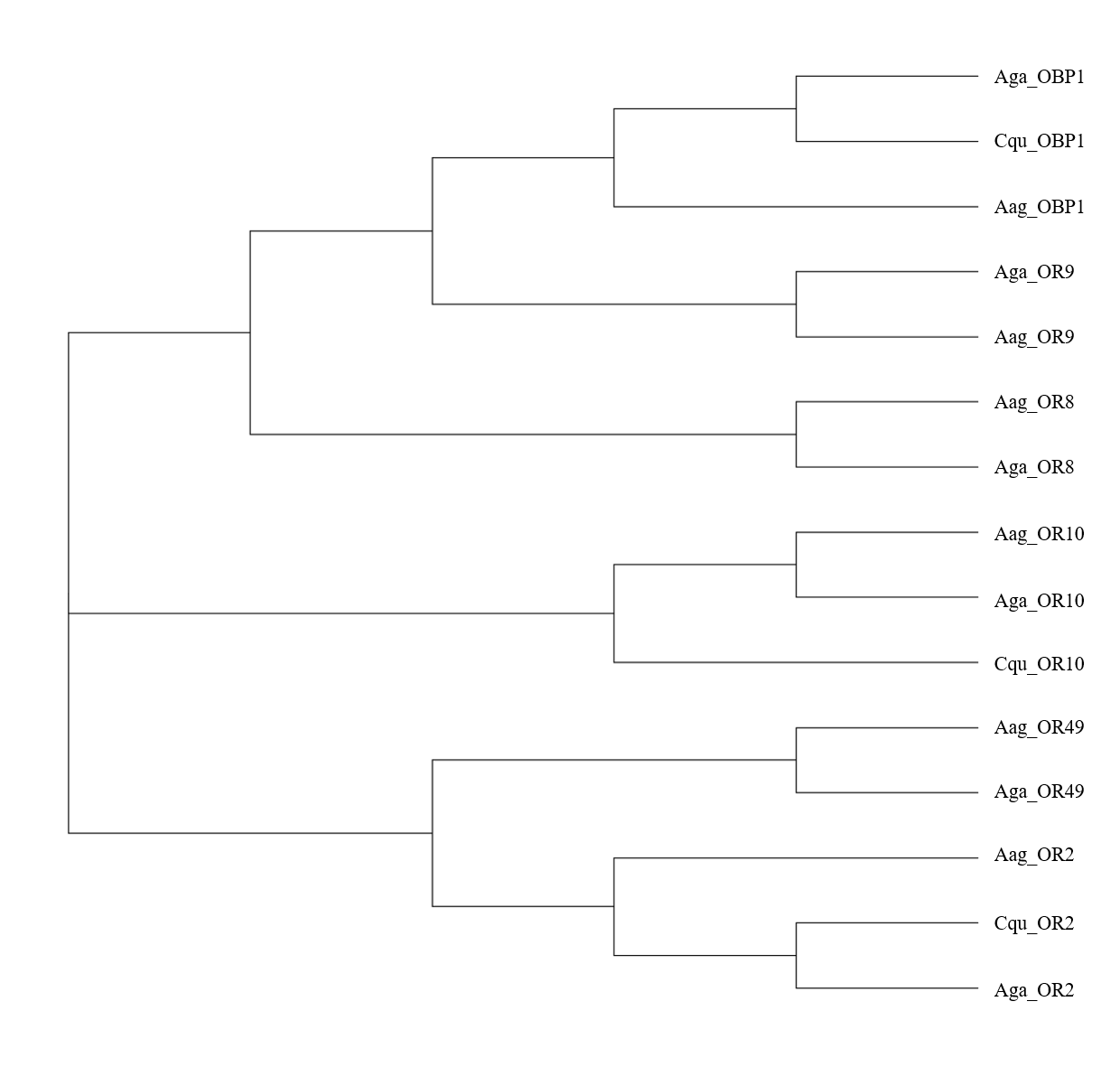

Supplement: S4 Fig — Aga stands for Anopheles gambiae, Cqui stands for Cx. quinquefasciatus, Aag stands for Aedes aegypti. Phylogenetic tree was constructed by the maximum likelihood (ML) method using Molecular Evolutionary Genetics Analysis (MEGA) 7.0 software, with 1,000 bootstrap replicates. (TIF) [file pntd.0010204.s004.tif]
