# Supplementary material for: Screening of olfactory genes related to blood-feeding behaviors in Culex pipiens quinquefasciatus and Culex pipiens molestus by transcriptome analysis
Source: PLoS Negl Trop Dis. 2022 Feb 7;16(2):e0010204. doi: 10.1371/journal.pntd.0010204 (PMC8853563; doi:10.1371/journal.pntd.0010204)
Supplement: S1 Table — Table A: The qPCR primers designed to verify olfactory genes; Table B: The primers used to synthesize dsRNA. (DOCX) [file pntd.0010204.s006.docx]

**Table A. The qPCR primers designed to verify olfactory genes**

| Gene name | Gene description | Primer sequence  (5'-3') | Product length  (bp) |
| --- | --- | --- | --- |
| RPL8 | House-keeping gene | F: AGTTCAAGCTCCGCAAGCA  R: CACGAACTGGCCGGTGTAC | 62 |
| 18S | House-keeping gene | F: ATTACGTCCCTGCCCTTTGTAC  R:CACCTTCAAAGACCTCACTAAATAATCC | 74 |
| CPIJ000545 | OR5 | F: AGTTAGCATTCTTAGTTCCGAT  R: GAAGCACCAAGAATACTCCAC | 128 |
| CPIJ004160 | OR13a | F: CAGATTTGCCACCTTCCGTTG  R: ATAGCACCATCACGTTAACCAG | 118 |
| CPIJ008793 | OBP56a | F: AAAAGGCGAAATAAGTCTGT  R: TTGTATCCAGCCTGTGCATC | 120 |
| CPIJ002105 | OBP58c | F: GCCAACGGTATGATAAATCGG  R: GGCTTCAATCTCGTCCATC | 144 |
| CPIJ016951 | OBP19a | F: GCAAGCTACCAAACGAGG  R: GCCCTGAGCTACATCCTT | 127 |
| CPIJ005920 | OR83c | F: CGTCTCTATCGCCAGTACCAG  R: AACAAACATCGTTGACCCGTA | 123 |
| CPIJ001730 | OBP4 | F: AACTCAAGGAACGAGCTG  R: TTGAGGCACTTTAATCCCAT | 95 |
| CPIJ016948 | OBP56e | F: CAAACTGTGTGCTCGAGA  R: TGGCTCAGCGATATCCTC | 107 |
| CPIJ002111 | OBP50d | F: ATCTGATTTCAAGCCCGTC  R: GTTCATCAGATAATACGGTGCAA | 124 |
| CPIJ008779 | OR83 | F: CTTTCATCCTGTATCTTTTGACGG  R: GCTGAAGCGAAATTACCTCC | 117 |
| CPIJ004162 | OR36 | F: AACATTTATCTGAGTGCGTTG  R: TGTAGTGCGAATCAGATCGT | 132 |
| CPIJ012950 | OR94b | F: CTGCCATTTGGATATTACTTTCCG  R: AAATCCGAGGCTTGTAACCCA | 122 |
| CPIJ006217 | OR65 | F: GCCCATTCATGTTGACGTT  R: TAACCAAACCACGAGTTACGG | 91 |
| CPIJ013644 | OR110 | F: TCAACGTCGTATGCTATCCG  R: TGAAAGTCTTCACCGAGTACGAG | 127 |

**Table B. The primers used to synthesize dsRNA**

| Gene name | Primer sequence(5'-3') | Product length (bp) |
| --- | --- | --- |
| EGFP-dsRNA | F: taatacgactcactatagggCAGTGCTTCAGCCGCTAC  R: taatacgactcactatagggGTTCACCTTGATGCCGTTC | 287 |
| OR5-dsRNA | F: taatacgactcactatagggCGTGCTGTATACGCTCGGTA  R: taatacgactcactatagggGGGGTTATTGCTAACGCAAA | 484 |
| OR78-dsRNA | F: taatacgactcactatagggTGCTACAACCAAATGTCCCA  R: taatacgactcactatagggTCCGAATCAACTGACACCAA | 490 |
| OR83-dsRNA | F: taatacgactcactatagggACCTCCTCGGACTCCACTTT  R: taatacgactcactatagggAGCTTAACCATAGCGGCTGA | 491 |

Note: The T7 promoter sequence is underlined.
